# Characterization of complete mitochondrial genome of two-spot swimming crab *Charybdis bimaculata* (Miers, 1886)

**DOI:** 10.1080/23802359.2018.1501292

**Published:** 2018-08-17

**Authors:** Nack-Keun Kim, Sapto Andriyono, Ah Ran Kim, Chung Il Lee, Hyun-Woo Kim

**Affiliations:** a Interdisciplinary Program of Biomedical, Mechanical and Electrical Engineering, Pukyong National University, Busan, Republic of Korea;; b Department of Marine, Fisheries and Marine Faculty, Universitas Airlangga C Campus Jl, Mulyorejo Surabaya East Java, Indonesia;; c Department of Marine Bioscience, Gangneung-Wonju National University, Gangneung, Republic of Korea;; d Department of Marine Biology, Pukyong National University, Busan, Republic of Korea

**Keywords:** Next generation sequencing, *Charybdis bimaculata*, mitogenome, decapod, phylogeny

## Abstract

The two-spot swimming crab *Charybdis bimaculata* (Miers, 1886) is an important decapod species in the benthic ecosystem of Korean waters. In this study, we determined its complete mitochondrial genome by the combination of NGS analysis using MiSeq platform and PCR-based cloning method. The circular mitochondrial genome of *C. bimaculata* was 15,714 bp in length in which the standard set of 13 protein-coding genes, 22 tRNA genes, and 2 rRNA genes were encoded. Phylogenic analysis showed that *C.bimaculata* is most closely related to *Charybdis feriata*. The complete mitogenome sequence information of *C. bimaculata* would provide useful data for the conservation of their population in the Pacific ocean.

*Charybdis bimaculata* (Miers, 1886) is a small portunid crab, which is mainly caught in Korea, China, and Japan (Rho et al. 2005; Gomez et al. 2008; Narita et al. 2008; Kwak et al. 2014). Although it is widely distributed and plays an important role in the benthic ecosystem (Kume et al. 1999; Yamaguchi and Taniuchi 2000), ecological study of *C. bimaculata* has been limited mainly due to its lack of genetic information. We here determined the complete mitochondrial genome of *C. bimaculata* using next generation sequencing (NGS) platform.

The specimen was collected from Jindo Island (34°22′42.62″N and 126°8′14.71″E), morphological identification was determined by National Institute of Fisheries Science, Korea. The specimen or its DNA was stored at Pukyong National University. Two mitochondrial DNA fragments were amplified by two degenerated primer sets targeting COI-ND5 and ND5-COI regions, respectively, and D-loop region was amplified by a sequence-specific primer set. Three PCR products were then pooled together in equal concentration and fragmented into smaller fragments (∼350 bp) by Covaris^®^ M220 Focused-Ultrasonicator^TM^ (Covaris Inc., U.S.A). Library was constructed by TruSeq® RNA library preparation kit V2 (Illumina, USA). After confirming the quality and the quantity using 2100 Bioanalyzer (Agilent Technologies, Santa Clara, CA, U.S.A), DNA sequencing was performed by MiSeq sequencer (2 × 300 bp ends) (Illumina, U.S.A).

The complete mitochondrial genome of *C. bimaculata* (GenBank Number: MG489891) was 15,714 bp which encodes 13 proteins, 22 tRNAs, 2 rRNAs and a putative control region (D-loop). Among them, 12 genes were initiated by the start codon ATN (ATG and ATT) with an exception of ATP8 gene, which begins with GTG. The canonical stop codons (TAA or TAG) were shown in nine genes (ATP8, ATP6, ND2, ND3, ND4, ND4L, ND5, and ND6), whereas incomplete stop codons (T–– or TA–) were found in the other four (COI, COII, COIII, and Cyt b) genes. Twenty one tRNA were predicted to be folded into a typical clover-leaf secondary structures by ARWEN (Laslett and Canbäck 2007) except for tRNA^Ser^, which was predicted without DHC arm.

The phylogenetic position of *C. bimaculata* within portunid crabs was analyzed using minimum evolutionary (ME) methods (Figure 1). As the result of phylogenetic tree which was constructed with the full mitochondrial genome sequences from 12 portunids and one Geryonid as out group, all four *Charybdis* species including *C. bimaculata, C. feriata* (Ma et al. 2015)*, C. natator* (Yang et al. 2017), and *C. japonica* (Liu and Cui 2010) were clustered together. *C. bimaculata* was most closely related to *C. feriata* showing 86% sequence identity (Figure 1). This information would be useful for extending our knowledge about evolutional relationship of Portunidae species.

**Figure 1.**
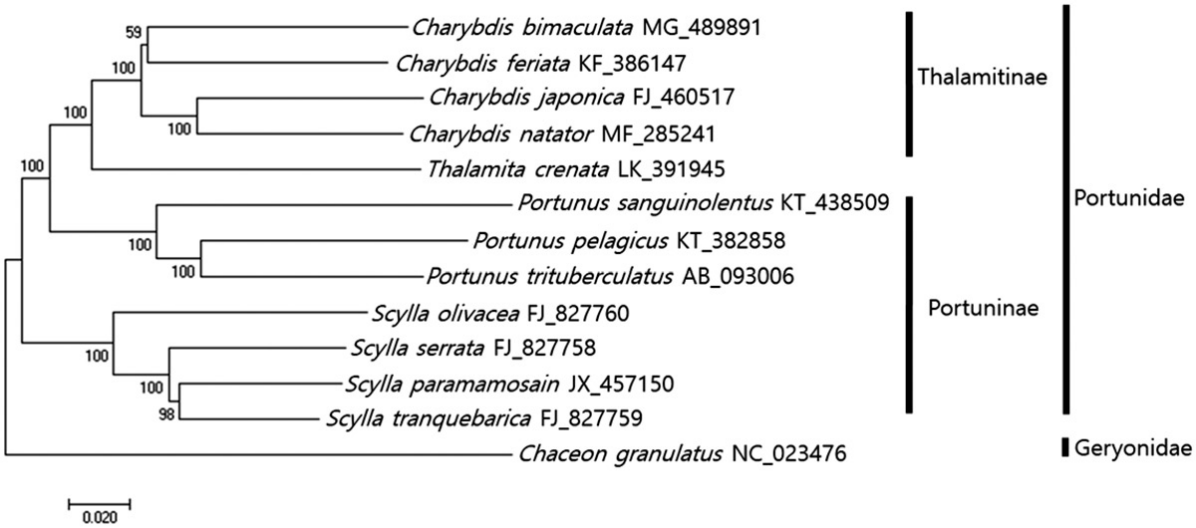
Phylogenetic tree of *Charybdis bimaculata* with other Portunid crabs. Phlyogenetic tree of *Charybdis bimaculata* was constructed with mitochondrial genomes of its relative species using MEGA7 software with Minimum Evolution (ME) algorithm with 1000 boothstrap replications. *Chaceon granulatu*s in Geryonidae was adopted as the out group member. GenBank Accession numbers were shown followed by each scientific name.
